# A U-shaped relationship between nighttime sleep duration and new-onset emotional, nervous, or psychiatric problems: a prospective cohort study from China

**DOI:** 10.3389/fpsyt.2025.1609865

**Published:** 2025-07-28

**Authors:** Hao Wu, Shuzheng Wang, Bingshuang Zhao, Haosheng Ni

**Affiliations:** ^1^ Department of Otolaryngology, Affiliated Hospital of Nantong University, Medical School of Nantong University, Nantong, Jiangsu, China; ^2^ Department of Otolaryngology, Rugao Branch Institute, Affiliated Hospital of Nantong University, Nantong, Jiangsu, China

**Keywords:** sleep duration, ENP(new-onset emotional, nervous, or psychiatric problems), CHARLS, mediating effect, prospective cohort study

## Abstract

**Introduction:**

Sleep plays an important role in maintaining physical and mental health, and it is important to study the relationship between sleep duration and new-onset emotional, nervous, or psychiatric problems (ENP).

**Methods:**

A prospective cohort study was performed based on data from Wave 2011, 2013,2015 and 2018 in the China Health and Retirement Longitudinal Study (CHARLS) databases. Sleep duration was assessed by self-reported nighttime sleep and daytime nap duration. Self-reported diagnoses were used to identify the new-onset emotional, nervous, or psychiatric problems (ENP). We used different logistic regression models to explore the potential effects of sleep duration on ENP and performed mediation analyses to assess the mediating roles of BMI, hypertension, and diabetes(DM).

**Results:**

Among 10,225 participants, 221 (2.16%) developed ENP during follow-up. The mean nighttime sleep duration was significantly shorter in the ENP group than in the non-ENP group. A restricted cubic spline regression model revealed a U-shaped relationship between nighttime sleep duration and ENP risk, with the lowest risk at 6.5 hours. Mediation analyses showed that BMI, hypertension, and diabetes did not significantly mediate this association (P values for ACME were all greater than 0.05).

**Conclusion:**

A U-shaped association was observed between nighttime sleep duration and new-onset ENP, suggesting that both insufficient and excessive sleep may increase the risk of ENP. These findings highlight the importance of maintaining an optimal sleep duration for mental well-being.

## 1 Introduction

Anxiety and other mental health problems are prevalent worldwide and have become a major public health challenge (1, 2). Recent statistics show that one in four to five people suffer from common mental illnesses, and 29.2% have experienced an episode of a common mental disorder in their lifetime (3, 4). Meanwhile, the overlap of somatic symptoms, anxiety, and depressive symptoms are common in the general population (3). It is well known that sleep plays a crucial role in maintaining physical and mental health (5). Sleep deprivation is recognized as a common symptom and risk factor for a variety of psychiatric disorders and is strongly associated with anxiety and mood disorders (6). Sleep deprivation can lead to a variety of adverse outcomes, such as insomnia that can lead to depressive symptoms, suicidal ideation, metabolic syndrome, and unexpected work-related deaths (7). Sleep deprivation can significantly impair an individual’s emotional regulation, leading to an amplification of negative emotional experiences (such as anxiety, depression, and irritability) and a reduction in positive emotional experiences (8). Sleep deprivation is also considered a significant risk factor in the onset and development of anxiety symptoms (9). Long-term sleep deprivation itself is an important risk factor for the onset of depression and may exacerbate existing depressive symptoms (10). Therefore, studying the impact of sleep duration on emotional, nervous, or psychiatric problems (ENP) is of great significance.

However, previous studies have mostly employed cross-sectional designs or short-term experimental sleep deprivation (11), making it difficult to reveal the long-term dynamic relationship and causal link between sleep and ENP. Therefore, there is an urgent need to design rigorous prospective cohort studies for further investigation.

To the best of our knowledge, no studies have reported on the relationship between sleep duration and new-onset ENP in Chinese middle-aged and elderly populations. Therefore, the present study aimed to investigate the potential association between the two and to provide scientific evidence and feasible suggestions for reducing the incidence of ENP from the perspective of sleep duration.

## 2 Materials and methods

### 2.1 Study population

This study used data from the China Health and Retirement Longitudinal Study (CHARLS) database (https://charls.pku.edu.cn/), a nationally representative survey established in 2011 to assess the aging population in China. The CHARLS project utilizes multistage stratified probability sampling and collects detailed demographic, health, and psychosocial information through face-to-face interviews. The cohort includes individuals aged 45 and older, making it an ideal dataset for examining the temporal relationship between sleep duration and the onset of emotional, nervous, or psychiatric problems (ENP) in middle-aged and elderly populations.

We performed a prospective cohort analysis using data from four waves: 2011 (baseline), 2013, 2015, and 2018. Participants were included if they had available sleep and ENP data and no ENP at baseline (2011). Those lost to follow-up or with incomplete covariate data were excluded. The baseline sample consisted of 10,225 participants, with an overall retention rate of 80.5% for the first wave (12). This high retention supports the robustness and internal validity of the study design.

**Figure 1:**
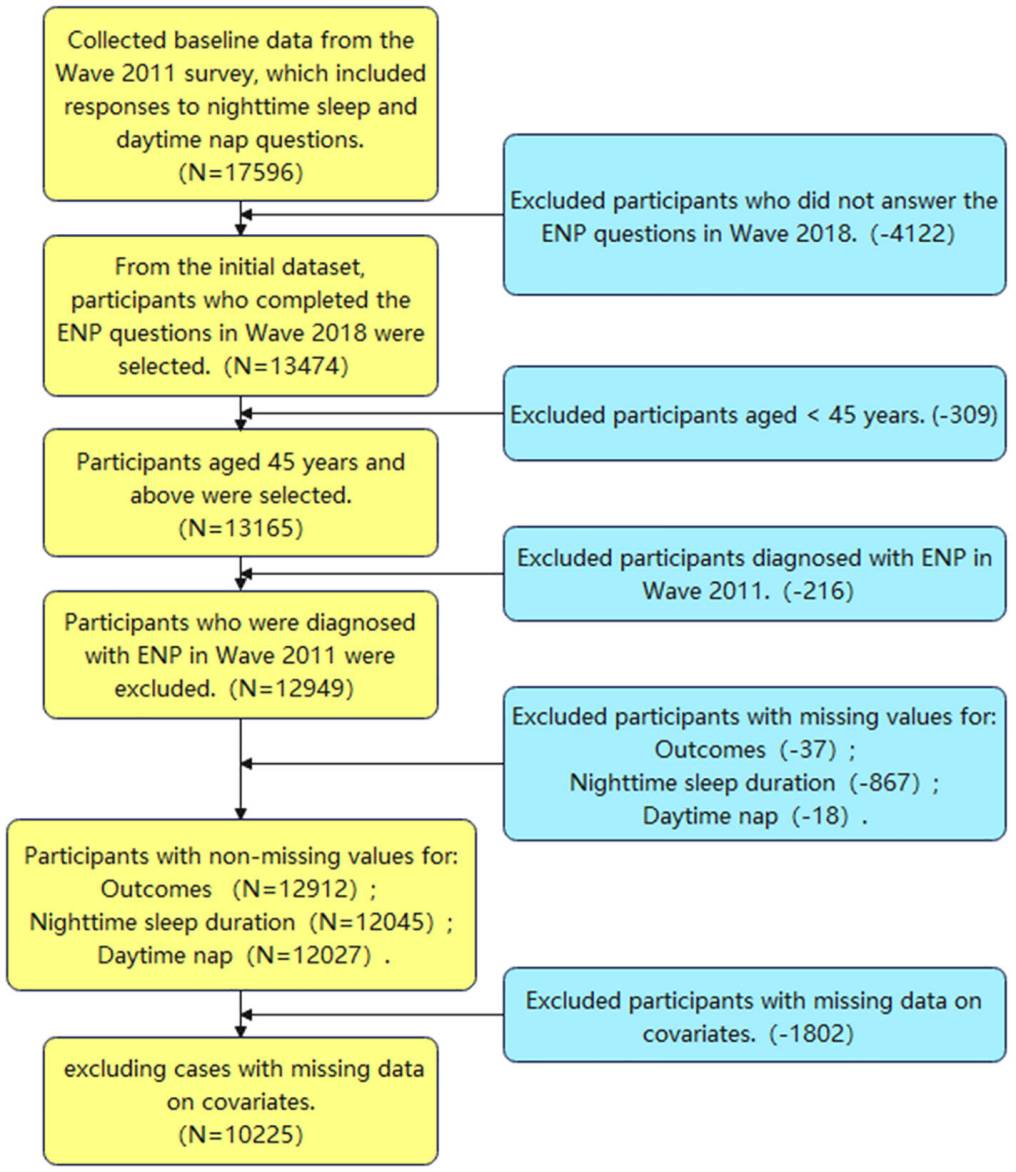
Flowchart of participant selection.

**Figure 1** illustrates the screening process, starting with an initial inclusion population of 17,596. Following the process, we excluded 7,371 individuals, culminating in a final baseline data population of 10,225.

### 2.2 Sleep duration assessment

Sleep duration (hours) includes both nighttime sleep and daytime nap, as reported by respondents in the self-report questionnaire. The nighttime sleep duration (hours) was assessed using the question “DA049. During the past month, how many hours of actual sleep did you get at night (average hours for one night)?”. The daytime nap (minutes) was assessed based on responses to the question “DA050. During the past month, how long did you take a nap after lunch?”

### 2.3 Assessment of new-onset ENP

New-onset ENP was diagnosed using self-report data DA007 (11). When the interviewer inquired, “Have you received a diagnosis from a doctor for emotional, nervous, or psychiatric issues?” and the respondent responded in the affirmative, we recognized them as having an ENP. We eliminated subjects who received an ENP diagnosis in 2011, but included individuals diagnosed with an ENP after that until the 2018 follow-up period, according to our criteria for a new-onset ENP.

### 2.4 Assessment of covariates

Socio-demographic characteristics such as age, gender, marital status, education level (primary, below high school, or university and above) and place of residence (rural/urban) were included in this study. Lifestyle factors included smoking and alcohol consumption status. Current diseases included hypertension and diabetes. In addition, different weight statuses defined according to body mass index (BMI) criteria were important covariates, including underweight (BMI < 18.5), healthy weight (18.5 ≤ BMI < 24), overweight (24 ≤ BMI < 28), and obesity (BMI ≥ 28), which we considered categorical variables in our stratified analyses, but for logistic regression and mediation analyses, BMI was treated as a continuous variable.

### 2.5 Statistical analysis

Continuous variables are expressed as mean ± standard deviation (SD) and categorical variables as percentages. We used multivariate logistic regression analysis to investigate the association between sleep duration and new-onset ENP in depth. We used three models to calculate the ratio (OR) and 95% confidence intervals (CI) of the association between sleep duration and the risk of new-onset ENP: crude model, an unadjusted model; model 1, which was adjusted for sociodemographic variables including age, gender, education, location of residence, BMI, and marital status; and model 2, which was further adjusted for the confounding factors such as smoking status, alcohol consumption status, hypertension, and DM. We also conducted interaction analyses to explore the effects of covariate factors on the sleep-ENP association and restricted cubic spline analysis to visually elucidate the dose-response relationship between sleep duration and the risk of new-onset ENP. Data analysis was performed with R software (Version 4.2.2).

## 3 Results

### 3.1 Characteristics of the study population

Subgroups were compared in this study based on the presence or absence of ENP (221 with ENP (2.16%) and 10,004 without ENP). The overall sample size was 10,225 people, and there were significant differences between the diseased and non-diseased groups in terms of age, gender, education level, alcohol consumption, and hours of sleep (**Table 1**). First, the mean age of the diseased group was significantly higher than that of the non-diseased group (59.50 ± 8.56 *vs*. 58.29 ± 8.77, P = 0.04). There was also a difference in gender composition, with 65.61% females in the diseased group, which was significantly higher than 53.31% in the non-diseased group (P < 0.001). In addition, in terms of education level, the proportion of those who received high school and below education was significantly higher in the diseased group than in the non-diseased group (76.47% *vs*. 67.92%, P = 0.02), whereas the proportion of those who received university and above education was lower. Smoking status showed a tendency to be significantly different between the two groups (P = 0.05), and drinking habits also showed a significant difference, with a significantly higher proportion of those who had been drinking for more than 1 month in the diseased group than in the non-diseased group (17.19% *vs*. 25.30%, P = 0.01). Regarding sleep duration, the mean sleep duration was shorter in the diseased group (5.98 ± 2.21 hours *vs*. 6.39 ± 1.84 hours, P < 0.01), but there was no significant difference in nap time (P = 0.99). Hypertension and diabetes mellitus (DM) were both higher in the prevalent group, but did not reach significant levels (P = 0.07 and P = 0.09). Finally, BMI was not significantly different between the two groups (P = 0.79).

**Table 1:** Baseline characteristics of the study population by new-onset ENP status.

	Total (n=10225)	No (n=10004)	Yes (n=221)	P value
**Age**	58.31 ± 8.77	58.29 ± 8.77	59.50 ± 8.56	**0.04**
Gender				<0.001
Female	5478(53.57)	5333(53.31)	145(65.61)	
Male	4747(46.43)	4671(46.69)	76(34.39)	
Marital status				0.32
Non-married	1109(10.85)	1080(10.80)	29(13.12)	
Married	9116(89.15)	8924(89.20)	192(86.88)	
Education				0.02
Elementary school and below	6964(68.11)	6795(67.92)	169(76.47)	
High school	3108(30.40)	3057(30.56)	51(23.08)	
College and higher	153(1.50)	152(1.52)	1(0.45)	
Residence place				0.95
Rural	6704(65.56)	6560(65.57)	144(65.16)	
Urban	3521(34.44)	3444(34.43)	77(34.84)	
Smoke				0.05
Never	6263(61.25)	6120(61.18)	143(64.71)	
Former	812(7.94)	788(7.88)	24(10.86)	
Current	3150(30.81)	3096(30.95)	54(24.43)	
Drink				0.01
No	6833(66.83)	6665(66.62)	168(76.02)	
<1 month	823(8.05)	808(8.08)	15(6.79)	
>1 month	2569(25.12)	2531(25.30)	38(17.19)	
Sleep duartion (hour)	6.38 ± 1.85	6.39 ± 1.84	5.98 ± 2.21	**<0.01**
Daytime nap(minute)	32.14 ± 42.46	32.14 ± 42.46	32.11 ± 42.79	0.99
Hypertension				0.07
No	6278(61.40)	6156(61.54)	122(55.20)	
Yes	3947(38.60)	3848(38.46)	99(44.80)	
DM(diabetes mellitus)				0.09
No	9044(88.45)	8857(88.53)	187(84.62)	
Yes	1181(11.55)	1147(11.47)	34(15.38)	
BMI	23.61 ± 3.89	23.61 ± 3.89	23.68 ± 4.04	0.79

### 3.2 Relationship between sleep duration and risk of new-onset ENP

**Figure 2:**
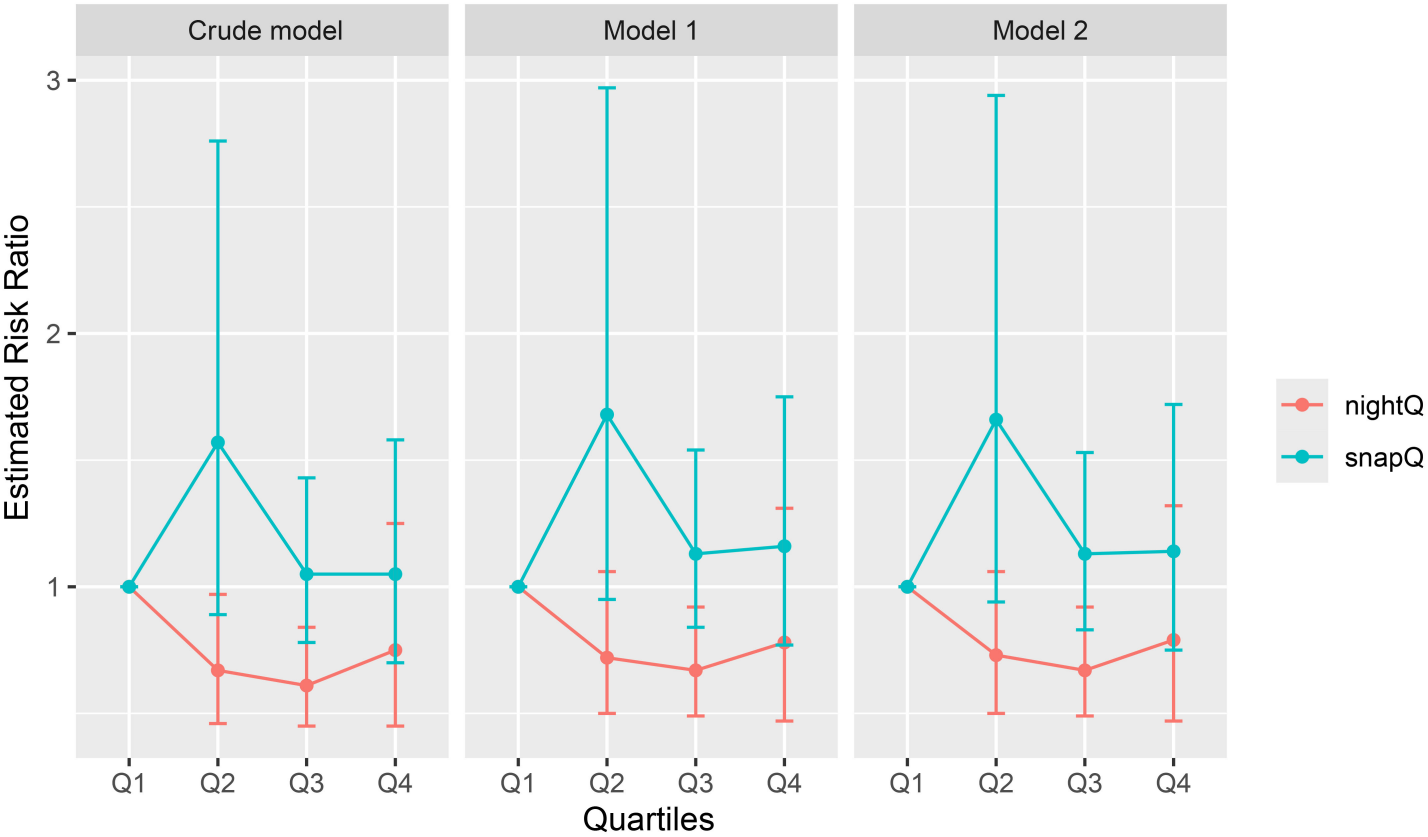
Effects of nighttime sleep duration quartile (nightQ) and nap duration quartile (napQ) on ENP in three different models (Crude model: an unadjusted model; Model 1: adjusted for age, gender, education, location of residence, BMI, and marital status; Model 2: further adjusted for smoking status, alcohol consumption status, hypertension, and DM).

Three models were used in this investigation to comprehensively examine the impact of sleep duration on the risk of new-onset ENP (**Table 2**, **Figure 2**). A substantial reduction in the risk of new-onset ENP was linked to longer overnight sleep duration in Crude, Model 1, and Model 2 (P<0.05). In particular, after controlling for all factors, the incidence of ENP decreased by almost 24% with every inter-quartile range (IQR) increase in sleep duration, OR = 0.76 (95% CI: 0.62, 0.94, p = 0.01). Using Q1 as the reference group, we evaluated the relationship between longer sleep duration and new-onset ENP after stratifying nocturnal sleep duration into quartile (Q1, Q2, Q3, Q4). Q3 showed a considerably reduced risk in all three models, and the trend test revealed a significant trend impact of sleep length in all three models (P<0.05), suggesting that the risk of ENP gradually decreased as sleep duration increased.

**Table 2:** Prospective associations between baseline sleep duration and daytime naps with follow-up new-onset ENP.

ENP	Crude model		Model 1		Model 2	
	95%CI	P	95%CI	P	95%CI	P
Sleep duration per IQR	0.71(0.58,0.88)	0.001	0.76(0.61, 0.93)	0.01	0.76(0.62, 0.94)	0.01
Q1	ref		ref		ref	
Q2	0.67(0.46,0.97)	0.04	0.72(0.50, 1.06)	0.09	0.73(0.50, 1.06)	0.10
Q3	0.61(0.45,0.84)	0.002	0.67(0.49, 0.92)	0.01	0.67(0.49, 0.92)	0.01
Q4	0.75(0.45,1.25)	0.27	0.78(0.47, 1.31)	0.35	0.79(0.47, 1.32)	0.37
P for trend		0.01		0.02		0.03
Daytime nap per IQR	1(0.83,1.21)	0.99	1.04(0.86, 1.26)	0.66	1.04(0.86, 1.25)	0.71
Q1	ref		ref		ref	
Q2	1.57(0.89,2.76)	0.12	1.68(0.95, 2.97)	0.08	1.66(0.94, 2.94)	0.08
Q3	1.05(0.78,1.43)	0.73	1.13(0.84, 1.54)	0.42	1.13(0.83, 1.53)	0.44
Q4	1.05(0.70,1.58)	0.80	1.16(0.77, 1.75)	0.49	1.14(0.75, 1.72)	0.54
P for trend		0.95		0.58		0.63

**Figure 3:**
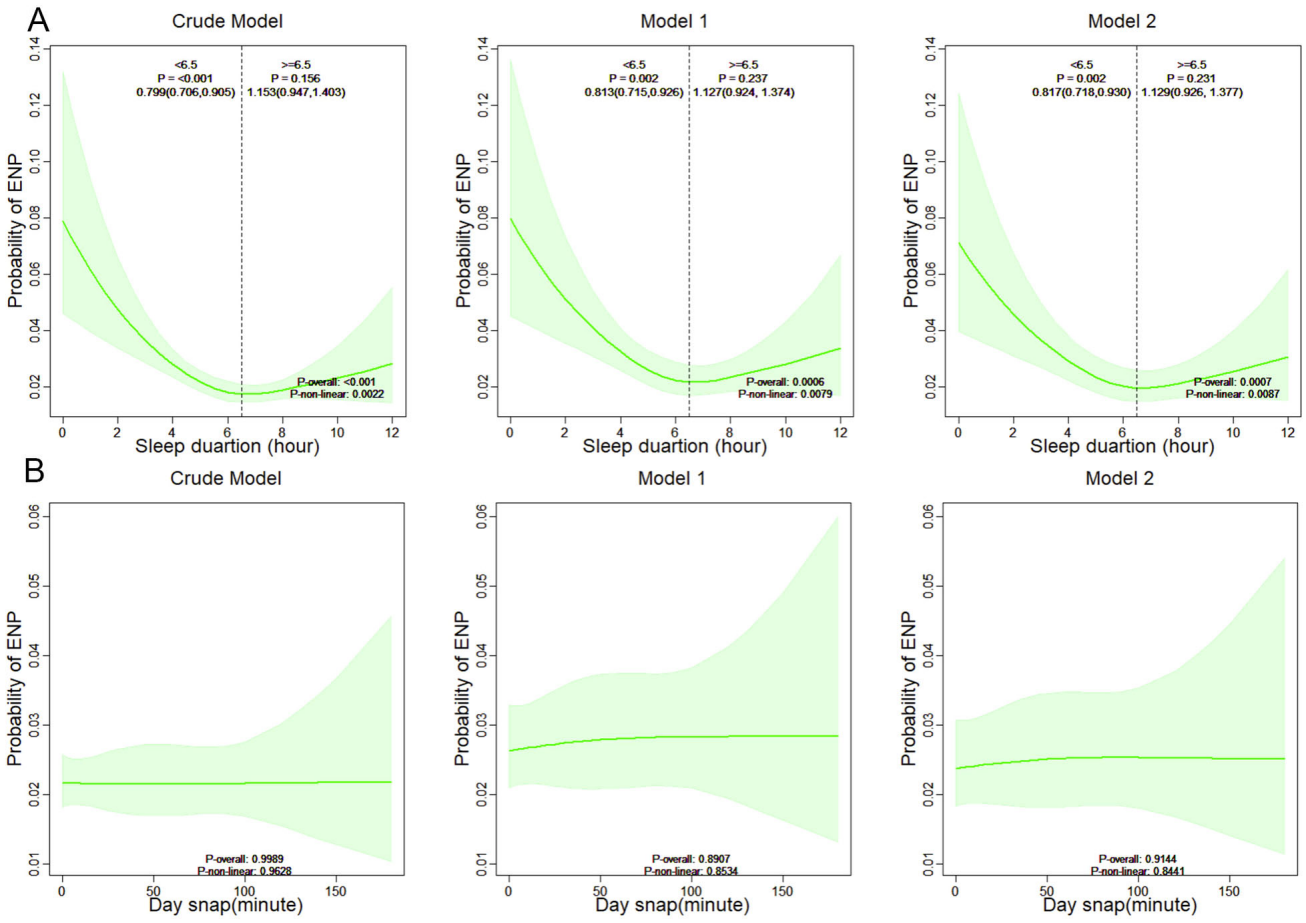
Association between sleep duration **(A)**, daytime nap **(B)** and risk of new-onset ENP in restricted cubic spline analysis.

The Restricted Cubic Splines (RCS) regression model was used to examine the link between overnight sleep duration and new-onset ENP risk (**Figure 3**). In all models, nighttime sleep duration and ENP risk showed a U-shaped curve relationship: shorter sleep duration (less than 6.5 hours) was associated with a significantly higher risk of ENP, and the risk gradually decreased with increasing sleep duration, reaching the lowest point after 6.5 hours, and then increased. At the ideal sleep duration (6.5 hours), new-onset ENP risk was the lowest.The relationship between sleep duration and new-onset ENP was significant (P-overall < 0.05) and non-linear (P-non-linear < 0.05) across all models (**Figure 3A**).

We also performed research on relationship between daytime naps and new-onset ENP, which found no significant connection and no significant trend across models and subgroups (**Figure 3B**). The P-value for each model was larger than 0.05 (P-overall > 0.8), and the non-linear test (P-non-linear) was likewise greater than 0.05, showing that there was no significant linear or non-linear relationship between daytime naps and new-onset ENP.

### 3.3 Subgroup analysis

**Figure 4:**
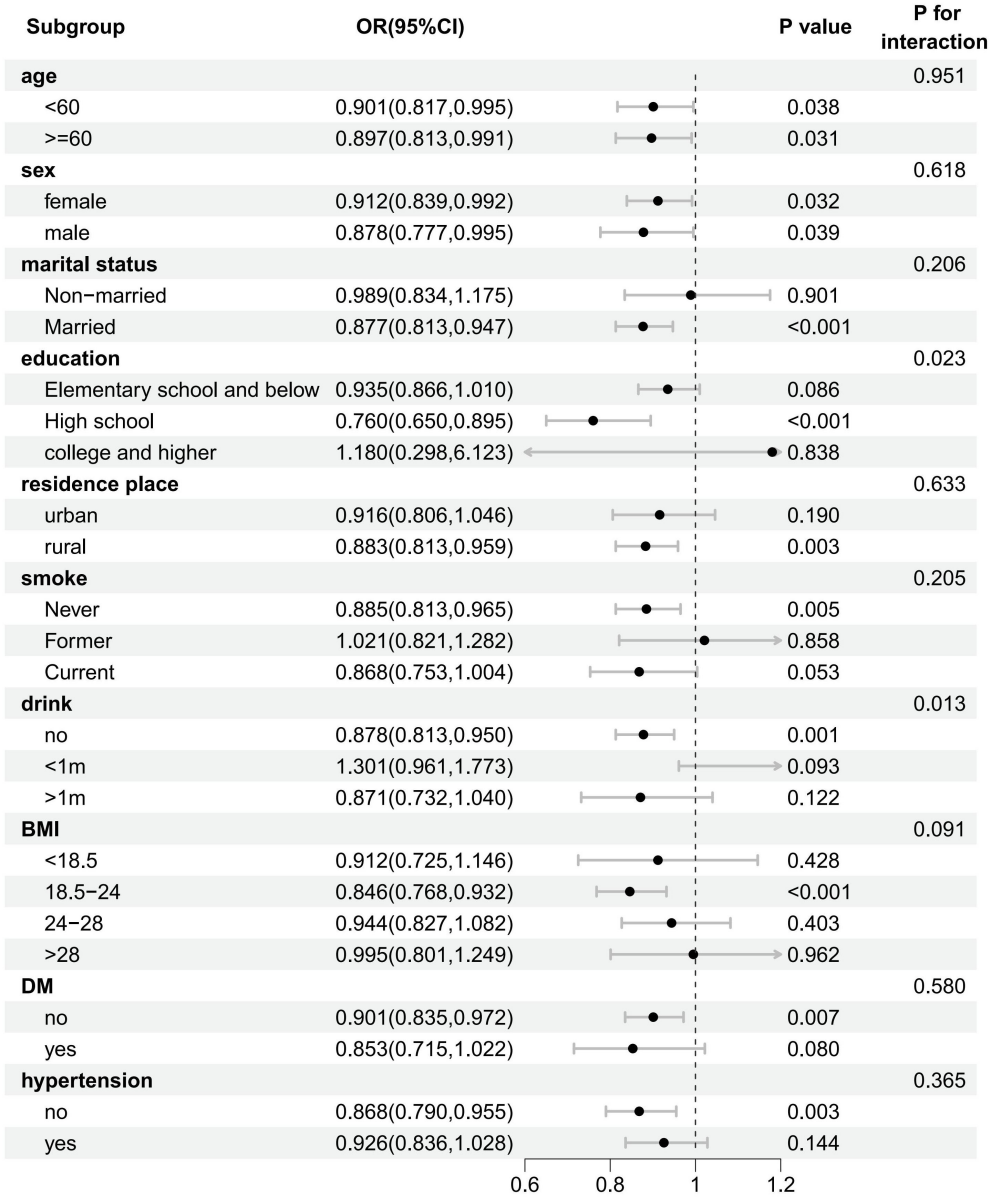
Forest plot of the stratified analysis of the association between sleep duration and risk of new-onset ENP.

Further subgroup analyses were conducted to investigate the variability of effects across groups, evaluate the stability of effects, and identify potential interactions (**Figure 4**). The results indicated that the risk of new-onset ENP was significantly reduced by increased nighttime sleep duration in the following subgroups: age, sex, married, high school education, living in the countryside, never-smokers, those with a BMI between 18.5-24, those without diabetes, and those without hypertension. The interaction analysis revealed that the effect of sleep on new-onset ENP was influenced by education and alcohol consumption factors.

### 3.4 Mediation analysis

In this study, we assessed the mediating effects of BMI, hypertension, and DM on the risk of new-onset ENP by mediation effect analysis of sleep duration at night (**Table 3**). The results showed that BMI, hypertension, and DM as mediating variables did not show significant mediating effects (P values of ACME were all greater than 0.05), suggesting that the indirect effects of sleep duration on ENP through BMI, hypertension, and DM were weak. However, the direct effect of nocturnal sleep duration on ENP was significant (all P values for ADE <0.05), indicating that nocturnal sleep duration was still significantly associated with new-onset ENP even after controlling for the effects of BMI, hypertension, and DM. Overall, the mediating role of BMI between nocturnal sleep duration and new-onset ENP was limited, and the effect of nocturnal sleep duration on the risk of new-onset ENP was mainly direct.

**Table 3:** Mediation effect of BMI, hypertension, and diabetes mellitus in the association between sleep duration and new-onset ENP.

	Effect	Estimate	95% CI Lower	95%CI Upper	P value
Sleep duration – BMI – ENP	ACME (average)	-0.000014	-0.00017	0.00012	0.844
	ADE (average)	-0.0031	-0.008	-0.00012	0.04
	Prop. Mediated	0.0044	-0.0634	0.092	0.838
Sleep duration – BMI – ENP	ACME (average)	-0.000023	-0.000091	0.000021	0.32
	ADE (average)	-0.0031	-0.0087	-0.00022	0.026
	Prop. Mediated	0.0074	-0.013	0.057	0.338
Sleep duration – BMI – ENP	ACME (average)	-0.000027	-0.00011	0.000017	0.284
	ADE (average)	-0.0032	-0.0082	-0.00019	0.026
	Prop. Mediated	0.0086	-0.01	0.054	0.304

## 4 Discussion

This study analyzed the association between total sleep duration (including nighttime sleep duration and daytime nap) and new-onset ENP using survey data from WAVE 2011, WAVE 2013, WAVE 2015, and WAVE 2018. After adjusting for the effects of covariates such as sex, age, BMI, hypertension and DM, the longer the total nighttime sleep duration, the lower the risk of ENP. Further analysis revealed a U-shaped relationship between nighttime sleep duration and the risk of new-onset ENP: When nighttime sleep duration was less than 6.5 hours, the risk of ENP gradually decreased with increasing sleep duration, reached a lowest point (6.5 hours), and then began to increase with further increases in sleep duration. This suggests that too little or too much sleep may increase the risk of ENP, and that an appropriate amount of sleep (6.5 hours) is ideal for mental health.

A substantial body of research has demonstrated the strong correlation between sleep and mental health, with sleep deprivation being identified as a significant risk factor for the development of mental health disorders (13). Adequate and high-quality sleep is not only essential for physiological recovery but also confers benefits with respect to cognitive function, mood regulation, immune system function and metabolic processes (14). During sleep, there is a notable increase in the convective exchange of cerebrospinal and interstitial fluids, which facilitates the removal of potentially neurotoxic waste products that may accumulate in the central nervous system during waking hours (15). The issue of sleep deprivation has been identified as a significant public health concern (16). Furthermore, research indicates that variations in sleep duration may be influenced by gender and ethnicity (17). The risk of adverse metabolic effects associated with sleep deprivation is also age-dependent (18). Nighttime sleep deprivation is a direct contributor to daytime sleepiness and impaired mood regulation, which in turn gives rise to more negative emotions and an increased risk of psychiatric disorders (19). From a mechanistic perspective, sleep deprivation may contribute to the development of age-related endothelial dysfunction, thereby accelerating the onset and progression of cardiovascular disease (20). Concurrently, diminished sleep duration is linked to elevated concentrations of growth hormone-releasing peptide, which influences energy metabolism by stimulating gastrointestinal motility and reducing insulin secretion (21). Furthermore, sleep deprivation may also serve to exacerbate the severity of mental health problems by increasing inflammatory responses (22). The results of the present study corroborate those of previous studies, confirming that a reduction in sleep duration is associated with an increased risk of new-onset ENP. In the present study, for each inter-quartile range (IQR) increase in sleep duration, the risk of psychiatric disorders was observed to decrease by approximately 29% (OR = 0.71, 95% CI: 0.58-0.88, P = 0.001). The present study also found, through further analysis, that an optimal sleep duration minimizes the risk of ENP, and in the present study this optimal sleep duration was 6.5 hours. Similar studies have previously provided support for this hypothesis. For example, Sun et al. identified a ‘U-shaped’ association between sleep duration and the risk of chronic kidney disease (CKD). Specifically, those who slept ≤4 or >10 hours per night exhibited an elevated risk of developing CKD compared to individuals who slept 6–8 hours per night (23). Similarly, Chen et al. found that an optimal sleep duration of 6–8 hours resulted in the lowest risk of developing atrial fibrillation compared to shorter or longer sleep duration (24).

The controversy over the relation between BMI and psychiatric disorders has not yet reached a consensus. Some studies have suggested that high BMI may affect mental health and emotional stability, and even depression, through biological mechanisms such as inflammation or endocrine dysregulation (25). However, some of the literature suggests that there is no significant association between BMI and mental disorders (26). The association between hypertension and mental disorders is also controversial. One study found that the prevalence of psychiatric disorders was significantly higher in hypertensive patients than in controls, with depression and anxiety being the most common symptoms (27). However, it has also been reported that there is no direct relationship between hypertension as part of metabolic dysfunction and depressed mood (28). The relationship between diabetes mellitus (DM) and ENP is similarly controversial. A cohort study of 1691 patients with type 2 diabetes showed that depressive symptoms may exacerbate diabetes distress, which in turn affects the severity of depressive symptoms (29). However, it has also been suggested that regardless of whether there is an association between diabetes and mental illness, the relationship may be confounded by socioeconomic status, lifestyle and genetic factors, among others. The development of mental health problems may not be a direct result of diabetes, but may be triggered by these common risk factors (30). In the present study, we explored the mediation roles of BMI, hypertension and diabetes in the relationship between nighttime sleep and the onset of ENP. The results of the mediation analyses showed that BMI, hypertension and diabetes did not have a significant mediating effect and that the effect of nighttime sleep duration on ENP was mainly direct rather than indirect through these covariates.

Furthermore, this study examined the influence of additional prevalent covariates on ENP, including age, gender, marital status, smoking, alcohol consumption, place of residence, and level of education. Among the factors exhibiting a significant effect were age, gender, education, and alcohol consumption. The results of our study indicate that females and older age groups are more susceptible to ENP symptoms. Additionally, individuals with lower levels of education may be at an elevated risk of developing ENP due to their exposure to life stressors and limited access to health resources. These findings are consistent with previously reported observations in the literature (31, 32). Additionally, our findings indicate an elevated risk of ENP among individuals who have never consumed alcohol. This observation contrasts with the majority of previous studies, which have concluded that alcohol consumption is significantly associated with psychiatric symptoms (33, 34). However, our results align with those of a survey study from rural China, which revealed a notable correlation between alcohol consumption and farmers’ mental well-being. Farmers who consumed alcohol exhibited a 19.7% higher mental health index compared to those who abstained from alcohol (35). We infer that the distinctive characteristics of the Chinese population may be the cause of these discrepancies.

Although the present study provides valuable insights into the relationship between nighttime sleep duration and new-onset ENP, it has several limitations. Firstly, the study primarily relied on participant self-reports for data, potentially leading to recall bias and compromising data accuracy. Secondly, this study did not measure sleep quality in detail, and future studies should incorporate objective sleep monitoring data to enhance data accuracy and further distinguish the effects of sleep duration and sleep quality. Thirdly, while we analyzed the effects of covariates such as BMI, hypertension, DM, smoking, and alcohol consumption and their mediating roles, we neglected to explore the underlying biological mechanisms, such as inflammation, neurotransmitters, or changes in hormone levels, that could significantly influence the relationship between sleep duration and the occurrence of ENP. Therefore, future studies should include the measurement of biomarkers to further elucidate the biological mechanisms of sleep duration on ENP.

## 5 Conclusion

Nighttime sleep duration showed a U-shaped relationship with the risk of new-onset ENP, with the most appropriate nighttime sleep duration (6.5 hours in this study) being associated with the lowest incidence of ENP. This finding provides new insights into our understanding of the relationship between sleep and mental health and highlights the need to consider sleep as an important intervention point when developing public health strategies and mental health interventions.

## Data Availability

The datasets presented in this study can be found in online repositories. The names of the repository/repositories and accession number(s) can be found in the article/supplementary material.
